# Assessing wear time and perceptions of wearing an ankle foot orthosis in patients with peripheral artery disease

**DOI:** 10.1002/pmrj.12829

**Published:** 2022-08-10

**Authors:** Danae Dinkel, Mahdi Hassan, John P. Rech, Holly DeSpiegelaere, Jason Johanning, Iraklis Pipinos, Sara Myers

**Affiliations:** 1 School of Health & Kinesiology, University of Nebraska at Omaha, Omaha, Nebraska, USA; 2 Department of Biomechanics, University of Nebraska at Omaha, Omaha, Nebraska, USA; 3 Department of Surgery and VA Research Service, VA Nebraska-Western Iowa Health Care System, Omaha, Nebraska, USA; 4 Department of Surgery, University of Nebraska Medical Center, Omaha, Nebraska, USA

## Abstract

**Background:**

Peripheral artery disease (PAD) is a cardiovascular disease that affects walking ability. An ankle foot orthosis (AFO) may improve walking distances in those with PAD. Little research has explored if those with PAD wear a prescribed AFO and their perceptions of wearing the device.

**Objective:**

To assess wear time of an AFO and explore perceptions of wearing the device in patients with PAD.

**Design:**

Convergent mixed methods.

**Setting:**

The study was conducted through a tertiary care medical center, and the research participants used the device in the community.

**Participants:**

Thirty-six patients, all older adult males, were enrolled in this study. Fourteen patients completed the study and 11 supplied sufficient accelerometer data to include in the analysis.

**Interventions:**

An AFO was worn for 3 months. An accelerometer was placed on the AFO for 7 days at the midpoint (1.5 months) and endpoint of the intervention (3 months) to assess wear time. Semi-structured interviews explored patients’ perceptions of wearing the AFO.

**Main Outcome Measure:**

The primary outcome measure was wear time measured objectively via accelerometer and subjectively via interview.

**Results:**

Patients (n = 14) wore the AFO approximately 8 hours/day. Patients reported barriers such as challenges wearing the AFO during daily household activities (using stairs, being on uneven terrain), discomfort, clothing or footwear issues, and driving challenges. Positive effects of wearing the AFO were also reported, primarily the ability to walk further.

**Conclusions:**

An AFO may be an acceptable therapeutic intervention to improve perceived walking performance in older adult males with PAD. Addressing participants’ perceptions of the AFO and barriers to wear are essential to increasing the positive effect the device has on participants’ ambulatory activity.

## INTRODUCTION

Peripheral artery disease (PAD) is a cardiovascular disease affecting more than 8.5 million Americans over the age of 40 years.^1^ PAD restricts blood flow to the legs and its most limiting symptom is intermittent claudication, which is pain or discomfort in the leg caused by physical activity and only relieved by rest. Participants with PAD have an ischemic myopathy in the skeletal muscles of their affected legs, which in conjunction with the low blood flow state limits walking performance from the first steps the participants take and prior to the onset of claudication pain.^2,3^ Participants with PAD are less physically active than individuals without PAD, taking an average of 3586 steps per day.^4,5^ Because patients with PAD are at an increased risk of cardiovascular events/mortality and participation in physical activity can combat this risk, efforts are needed to help patients with PAD be more physically active.^6,7^

A common nonpharmacologic approach to treatment of PAD is a supervised exercise program. Supervised treadmill exercise has been found to increase the distance participants with PAD can walk.^6^ However, barriers such as lack of insurance coverage, inconvenience, or travel/access difficulties, reduce participation in these programs.^6,8^ An ankle foot orthosis (AFO) has been proposed as a cost-effective assistive device that would decrease the muscular demand during walking. When made of carbon-composite materials, AFOs allow energy storage at weight acceptance that can be returned just before toe-off, when the ankle plantar flexors are supposed to push-off into the next step. The energy provided by the AFO at push-off allows a reduction in the contribution needed from the ankle plantar flexor muscles.^9^ Decreasing muscular demand reduces the energetic requirements of the myopathic muscles, which may enable people with PAD to walk further, or walk the distance needed, with less pain.^9^

For those with PAD to experience the potential benefits of the AFO, it must be worn during walking. Previous research has found poor adherence to wearing braces or other orthotic devices.^10-15^ For example, a study of participants with diabetes measured the ability to wear a device (removable cast walker) over 7 days. Only 30% of the participants were high utilizers (wore the device more than half the time) and, on average, these participants wore the device 60% of the time.^10^ Another study of individuals with Charcot-Marie-Tooth disease found that only 20% of participants were wearing the prescribed AFO outside of the house. In qualitative interviews, participants indicated that the AFO was uncomfortable, brought attention to their disability, wasn’t needed, and made it difficult to find shoes that worked with the device.^14^

Previous work examining the use of an AFO in participants with PAD has found that participants significantly increased their peak walking time and delayed the onset of claudication.^16^ In a qualitative study exploring participants’ perceptions, participants believed that the AFO improved their ability to walk and their quality of life, particularly when used on flat surfaces.^17^ However, they did experience barriers to wear such as walking on uneven ground, driving, and existing lower extremity conditions. The authors suggested that future qualitative work in different AFO designs are needed.^17^ Thus the objective of this study was to assess wear time of an AFO and explore perceptions of wearing the device in participants with PAD. We hypothesized that participants would have positive reactions to the AFO on their perceptions of their walking distance; however, they would experience barriers to wear including challenges with walking on uneven ground and driving.

## METHODS

### Participants

Inclusion criteria were (1) able to give written, informed consent; (2) Fontaine stage II PAD^18^; (3) demonstrate exercise-limiting claudication established by history and direct observation during a screening walking test administered by the evaluating vascular surgeon; (4) have an ankle/brachial index ≤.90 at rest^19^; and (5) have a stable blood pressure regimen, stable lipid regimen, stable diabetes regimen, and risk factor control for 6 weeks. Exclusion criteria were (1) rest pain or tissue loss due to PAD (Fontaine stage III and IV); (2) acute lower extremity ischemic event secondary to thromboembolic disease or acute trauma; and (3) walking capacity limited by conditions other than claudication, including leg (joint/musculoskeletal, neurologic) and systemic (heart, lung disease) pathology. Patients were referred to the study by their vascular surgeon at the Nebraska-Western Iowa Health Care System. Research personnel confirmed that the participants met the inclusion/exclusion criteria prior to scheduling their first visit. All participants provided written informed consent. This study was approved by the Nebraska-Western Iowa Health Care System and the University of Nebraska Medical Center Institutional Review Board and has been registered as a clinical trial (NCT02902211).

### Study design

**FIGURE 1:**
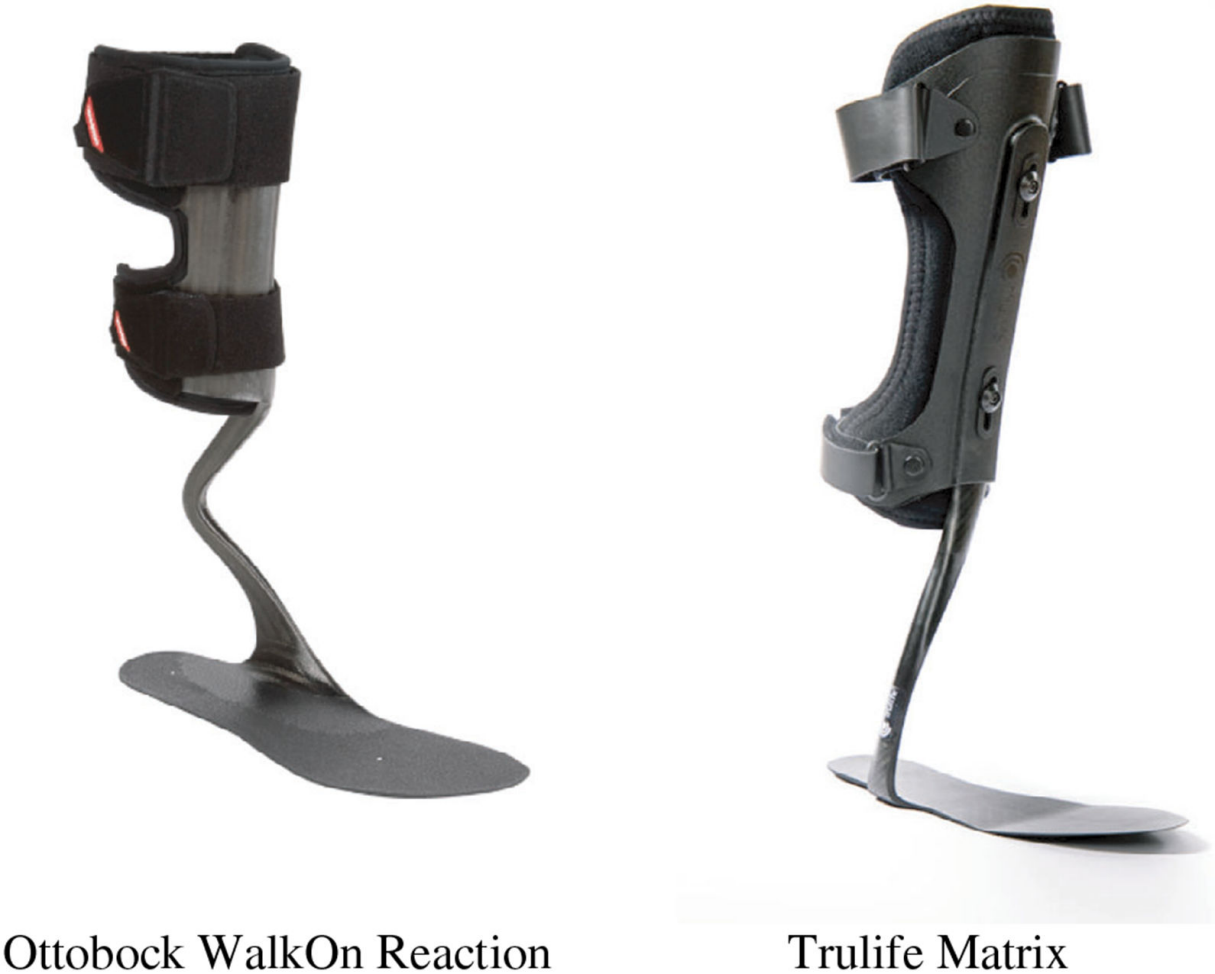
Ankle foot orthosis (AFO) options

The purpose of this prospective convergent mixed-methods study was to monitor wear time of an AFO and explore the perceptions of wearing the device to improve the wear time of future assistive devices.^20^ This study was part of a larger crossover design protocol examining the effects of an AFO on walking performance in patients with PAD (NCT02902211). Additional details on the full protocol can be found elsewhere.^21^ The present study consisted of having participants wear an AFO on both legs for 3 months. The study was administered through a tertiary care medical center, and the research participants used the device in a community setting, in and out of their homes, during their regular activities. Participants were fitted with one of two different carbon-composite AFOs (Figure 1) and accompanying shoes by a certified orthotist at the Nebraska-Western Iowa Health Care System. The two AFOs used for the study included the WalkOn Reaction AFO (Ottobock, Duderstadt, Germany) and Trulife Matrix (Trulife, Jackson, MI). The certified orthotist or prosthetist determined which AFO to use for each participant. These two devices were selected because they represent a carbon composite design that has been shown to reduce energy costs.^9^ Both devices have similar stiffness characteristics and amount of motion allowed. When participants were provided with the AFO, they were encouraged to wear the AFO as much as possible for the next 3 months. Data were collected from November 2017 to January 2020.

#### Outcome measures

##### Wear time by accelerometers

To assess wear time, at the intervention midpoint (1.5 months) and endpoint (3 months), participants were mailed a GT9X Actigraph accelerometer (Pensacola, FL) to be placed on the AFO. Participants were instructed to place the accelerometer only on the right AFO, at a predetermined location, using a provided three-dimensional (3D)–printed accelerometer holding device and to wear the accelerometer for 7 consecutive days. At the midpoint data collection period, participants mailed the accelerometer back to research personnel. After the endpoint data collection period, participants brought their accelerometer back with them to their final data collection appointment. A minimal wear time of 3 days was required to ensure sufficient data were supplied by participants.^22^

##### Wear time perceptions

To assess participants’ perceptions of their wear time, as well as positive and negative implications of wearing the AFO, semi-structured qualitative interviews were conducted via phone at midpoint and in-person at endpoint. If the researcher was not available for the in-person endpoint data collection, a phone interview was scheduled. Interviews that were conducted in-person occurred in the lab, in which doors were closed, with only institutional review board (IRB)–approved study personnel present. Interview questions followed Bowen and colleagues’ standards for feasibility studies.^23^ Specifically for wear time, we focused on the aspects of Acceptability (satisfaction of using the AFO) and Implementation (degree of use, success/failure of use, factors affecting the ease/difficulty of use). Please see Table 1 for an overview of questions. Interview questions were pilot tested with the first two participants and minor adjustments were made (i.e., reference to the AFO was changed to braces). A trained PhD associate professor (lead author) with 10 years of qualitative research experience conducted the interviews. The researcher and participant had not met prior to the midpoint phone call, and the call was scheduled by the study coordinator. At that time, the researcher introduced herself to the participant and explained the purpose of the interviews. Interviews lasted about 10 minutes.

### Data analysis

Wear time was calculated using the Actilife software’s wear time validation algorithm following the protocol from Choi and colleagues.^24^ Tests of normality revealed that the data were normally distributed as assessed by the Shapiro-Wilk’s test (range from *p* = .06 to *p* = .97). Descriptive data including the mean and standard deviation (SD) were calculated. A paired-samples *t*-test was performed to examine the differences between midpoint and endpoint for the variables: average total days and hours per day; average weekdays and weekday hours per day; and weekend days and weekend hours per day the AFO was worn. A two-tailed significance was set at the *p* < .05 level.

Qualitative data were transcribed verbatim into a MS Word document and then uploaded into QSR NVivo 12.^25^ A directed content analysis approach was used to analyze data.^26^ Codes were developed using a deductive approach, which develops codes using existing theory or frameworks by following the Bowen and colleagues constructs of Acceptability and Implementation.^23^ An inductive approach, in which codes are developed based on the commonalities between participant responses, was used to develop codes underneath these parent themes. Data were validated through the process of peer debriefing and interviewing to saturation.^27^ In addition, as the same questions were asked at both midpoint and endpoint data collection, the second interview served as a validation of the answers at midpoint. Because there were no major differences found between interviews within each participant, the midpoint interview was used in reporting results.

## RESULTS

**FIGURE 2:**
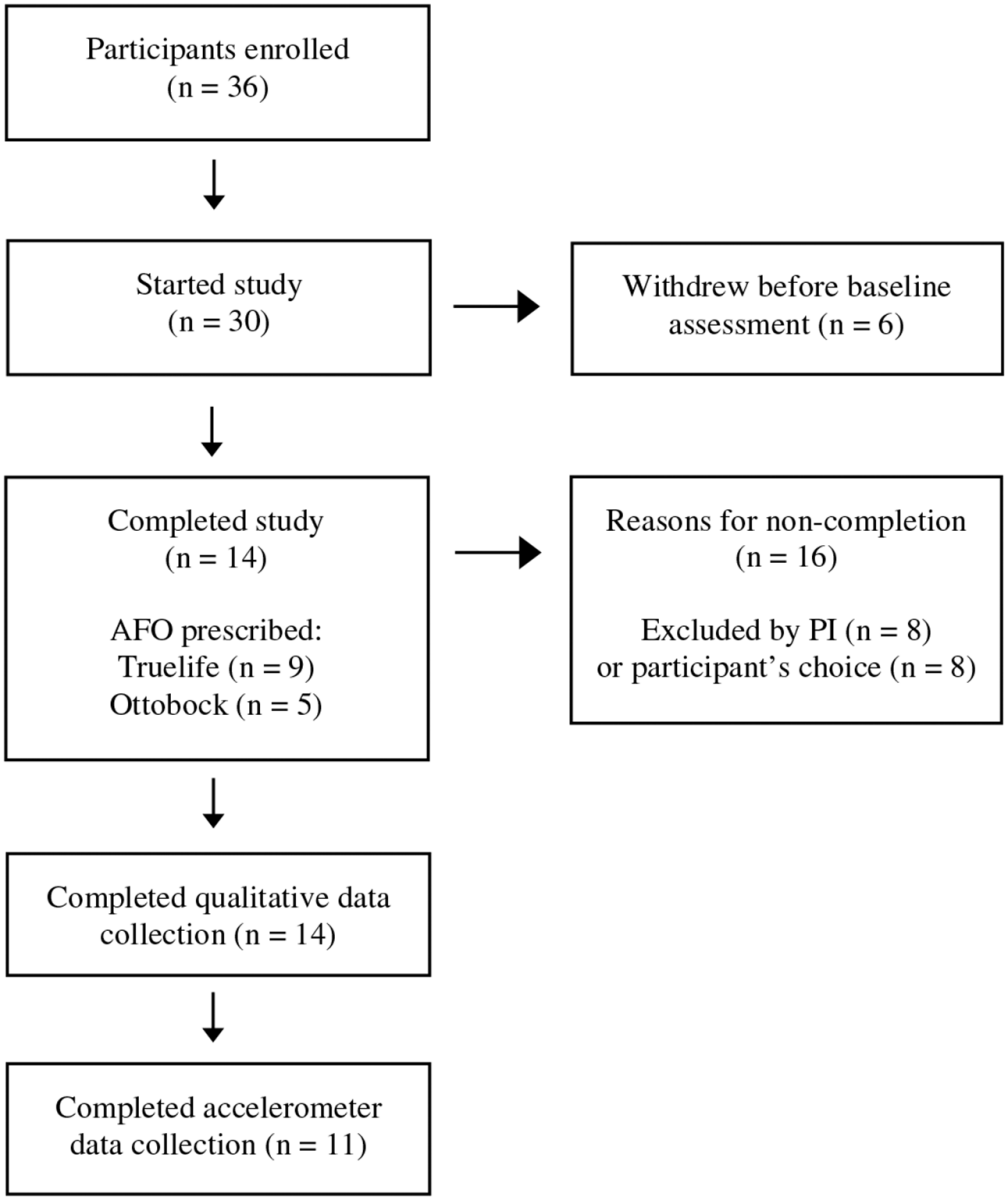
Flow diagram of participant retention

Thirty-six participants enrolled in the study. Six participants withdrew before baseline assessments due to reasons such as health conditions and living too far away from the study; and additional participants were withdrawn by the principal investigator (PI) (n = 8) or their own choice (n = 8). Thus a total of 14 participants completed the study. Of these 14 participants, accelerometer data were available for only 11 participants due to lack of sufficient data as these participants wore the device for less than 3 days at both time periods; however, all 14 participants were included in the qualitative data analysis (Figure 2). All participants were male; 92.9% were non-Hispanic White with an average age of 71.9 years (SD = 6.7) and body mass index of 29.0 (SD = 5.5). Participants reported that PAD pain was mixed with reports of pain in calf-only, thigh-calf, buttock-calf, and buttock-thigh-calf; however, all participants noted pain in their calf.

### Wear time by accelerometer

Table 2 provides the descriptive information regarding wear time. On average, individuals wore the AFO for 5 days per week and 8 hours per day. There were no significant differences between wear time at midpoint and endpoint interventions regarding total number of days worn (*p* = .49; 95% confidence interval [CI] −0.77 to 1.50), total hours per day (*p* = .54; 95% CI −1.35 to 2.43), number of weekdays worn (*p* > .99; 95% CI −1.00 to 1.00), number of hours worn on weekdays (*p* = .19; 95% CI −0.58 to 2.65), number of weekend days worn (*p* = .17; 95% CI −0.18 to 0.91), or number of hours per weekend day worn (*p* = .97; 95% CI −2.80 to 2.89). When comparing the number of hours the AFO was worn during week days and weekend days at midpoint there was a significant difference (*p* = .03; 95% CI 0.21 to 4.36) with the AFO being worn more often during the week (8.8 ± 3.9) compared to the weekend (6.5 ± 6.0). However, this difference was no longer there at the endpoint of the intervention (*p* = .24; 95% CI −1.00 to 3.60).

### Semi-structured interview findings

The findings present participants’ perceptions of wearing the AFO. The major themes identified were perceived wear time, barriers to AFO use, and positive effects. Overall, a majority stated that they wore the AFO most of the time; however, participants reported that they experienced barriers when wearing the AFO around their home, going up and down stairs, and while driving. Almost all participants reported that they felt the AFO positively influenced their ability to walk.

#### Perceived wear time

When participants were asked about the amount of time they wore the AFO, 71.4% felt they wore the AFO a majority of the time. Participants often referred to wearing the AFO only when they were outside of the house. For example, one participant mentioned, “I wear ‘em every day…I just don’t wear ‘em around home walking around. You know in the afternoon I’m getting ready to relax and take it easy, I take ‘em off.” The remaining participants only referred to wearing the AFO for a minimal amount of time as one participant mentioned, “…I don’t do it very often or very long.”

#### Barriers to AFO use

All but one participant mentioned barriers to wearing the device. The barrier reported most often was in relation to the challenge of wearing the AFO while they were doing daily activities throughout their house or around their home (78.6%). A majority of participants mentioned that the AFO was challenging to use while going up and down stairs: (64.3%) as one participant stated, “Going up and down stairs is really difficult because I can’t bend my ankle.” Another often reported barrier related to daily activities was the challenge of being outside on uneven terrain. Participants (35.7%) reported that trying to do activities outside of the house in their own yard could be challenging. For example, one participated reported “…if I get off (the sidewalk) and walk on grass somewhere then I really have a hard time.”

Discomfort, clothing or footwear issues, and driving challenges were also reported as top barriers to wear. Regarding discomfort, half of participants reported some type of discomfort such as on their shin or the bottom of their feet. Regarding clothing or footwear issues, participants (35.7%) were unsure how the AFO would fit with other shoes for different weather (e.g., snow boots) and did not like that they could only wear the AFO with certain clothes (e.g., certain types of pants). Finally, regarding driving, 42.9% of participants reported that while they were wearing the AFO they felt unsafe while driving. For example, one participant mentioned, “It’s hard to find the gas pedal and brake.”

Remaining barriers that were mentioned were the challenge of just being within the house. Many participants noted that they most often wore the device when they were going somewhere or were up and doing something. However, several participants (28.6%) noted that they would not wear the AFO if they were only going to be within the house. For example, one participant noted, “When I’m just sitting here I’m usually just sitting around with slippers or socks.” Furthermore, another portion of participants (21.4%) had other health-related issues that stopped them from wearing the device. One participant noted “I’ve had some medical problems and I’ve been in the hospital.…”

#### Positive effect of wear

Almost everyone (92.8%) mentioned that they felt positive effects from wearing the AFO. The primary perceived improvement reported by the participants was the ability to walk longer distances (78.6%). Participants mentioned that they noticed these changes in a variety of areas and often while doing daily activities. One participant stated “Like at best I could walk from the front to the back of Walmart before I would start to hurt. With the braces I was able to do all my grocery shopping.” Similar to the previously reported barrier, some participants (14.3%) specifically mentioned that they only felt these improvements when they were on “flat walkable ground.” In addition, one participant mentioned that the AFO improved their pain, “they stop my legs from hurting anymore.”

## DISCUSSION

The purpose of this study was to assess wear time of an AFO and explore the perceptions of wearing the device. Accelerometer data found that participants wore the AFO for about 5 days per week for ~8 hours per day. Almost all participants reported both barriers and positive experiences with the AFO. Results suggest that an AFO may be an effective therapeutic intervention to improve certain functional tasks such as tolerance to level-surface ambulation in patients with PAD.

Overall, patients wore the device for a little less than 5 days per week and 8 hours per day at both midpoint and endpoint of the intervention. The accelerometer data corresponds to the qualitative interviews where participants reported wearing the AFO a majority of the time, but mainly wearing the device outside of the house. Our previous work has found that patients with PAD were primarily active over a 10-hour period (8:00 am - 6:00 pm) obtaining an average of 3586 steps per day. In addition, patients spent a large amount of time during waking hours (on average 7 hours per day) being sedentary.^5^ Furthermore, most participants mentioned being retired within the interviews and the wear time may correspond with the amount of time they were doing activities that required putting on shoes—a necessity for wearing the AFO. A study examining the use of custom-made footwear for patients with diabetes also found higher compliance with wearing the footwear outside of the home.^28^ Given the limited amount of time previous research has found that participants with PAD were active during the day, the data from the present study support that participants wore the AFO for a majority of the time that they were active.^5^

Although a specific amount of time was not prescribed to patients in this study, based on the accelerometer data and the typical waking periods of older male adults, patients wore the AFO for ~50% of their waking hours, which corresponds to our previous work that patients spend half of their awake time in sedentary behavior.^5^ Studies in other patient populations have found poor adherence with wearing an AFO and other supportive devices.^10-13^ A systematic review examining participant compliance with wearing an orthotic device or shoe found that 6% to 80% of patients did not use the device.^15^ Studies specifically examining orthotic devices such as AFOs have found as little as 20% of participants report wearing the AFO.^14^ However, a majority of these studies were based on participant self-report questionnaires or interviews.^16^ Additional research is needed to objectively examine wear time of AFOs and other orthotic devices.

It is important to note that that there were no significant differences in wear time of the AFO between the midpoint and endpoint of the intervention. The only significant difference between wear time was on weekdays versus weekends at midpoint; however, this was no longer significant at endpoint. This could indicate that as people became more used to wearing the AFO, they were more likely to also wear it on the weekends. However, additional research is needed to better understand these findings. Furthermore, it may be important to objectively assess wear time earlier on in the intervention such as during the first week or two to help address wear time challenges (e.g., fit issues of the AFO) earlier on to better increase adoption of wearing the AFO as well as length of wear time.

Similar to other research, patients reported several barriers to wearing the AFO including that it was uncomfortable to wear and concerns of difficulties finding footwear.^11,17^ Choma and colleagues (2020) also examined the perspectives of participants with PAD who used the Ottobock WalkOn Reaction and reported that wearing the AFO was difficult on uneven ground and while doing activities around the house such as walking up and down stairs and doing yard work.^17^ Given the low physical activity of participants with PAD, ensuring that they can comfortably wear the AFO around their home is critical to increase adherence in order to improve physical activity levels.^4,5^ Contrary to our findings, a study by Mays and colleagues (2019), who examined the effect of the Ottobock WalkOn Reaction AFO during an unstructured community-based walking program, found that participants with PAD had self-reported improvements in their ability to walk up and down stairs after using an AFO for 12 weeks.^16^ Discomfort in general and challenges while driving were other important barriers reported by participants in our study, and others, that could greatly limit participant wear time of the AFO.^17^ Efforts are needed to address driving barriers to improve adherence to wearing the AFO by participants with PAD.

Regardless of wear time, almost all participants reported perceived benefits to wearing the AFO. Similar to other research of individuals wearing an AFO and specifically people with PAD, participants reported that they could walk farther and most often saw these differences while doing daily activities.^16,17,29^ The study by Mays and colleagues also found that participants with PAD who wore an AFO perceived improvements in their overall quality of life.^16^ Other research has found the most sought after features of AFOs related to effectiveness, reliability, durability, and comfort as well as ensuring that the AFO can help improve walking and standing.^11,29^ In addition, participants relied on the devices to enable them to pursue daily activities such as job duties, driving, outdoor activities, and social events. Future design efforts should emphasize comfort and improvements in ankle articulation in order to improve user ability to take part in daily activities such as home care, outdoor activities (lawn care), family events, and other related activities.^11^ Furthermore, an assistive device that could be worn inside the home without shoes would be particularly useful for those with PAD, given the amount of time they spend inside.

There were several strengths and limitations of this study. A primary strength of this study includes the mixed methods study design through use of both objective and subjective data. Another strength is that this is one of the first studies examining wear time of an AFO with participants with PAD. It is important to note that while several of our qualitative study findings were similar to other work,^16,17^ in which the use of the Ottobock WalkOn Reaction was explored with participants with PAD, our study used both the Ottobock and Trulife Matrix.^17^ There were several limitations. First, the wear protocol used for accelerometers in this study has not been validated previously to assess wear time of an AFO, and it only collected data from two 7-day data collection periods. Although this is a widely accepted data collection period in physical activity literature, future research should monitor wear time over a longer period to ensure that this is an accurate representation of wear time. The purpose of placing the accelerometer on the AFO was to determine if the device was in motion to determine wear time rather than the amount of physical activity; we believe this is an appropriate representation of wear time. Furthermore, the accelerometer data aligned with the qualitative data, providing further validation of the data. Future studies could validate wear time with a temperature sensor. Second, the population, being recruited from the local Veterans’ Affairs medical center, was primarily a White male population and thus may not be representative of all individuals with PAD. In addition, there was a high dropout rate (over 60%) of participants, resulting in a small sample size. Third, the distance ambulated was not objectively assessed and distance walking improvements are reported from interviews. Further, a baseline assessment of functional skills such as using the stairs was not included. Future research should examine objective assessments of physical activity and functional skills. Finally, a control group was not included. The inclusion of these aspects in future study designs could further elucidate wear time findings.

## CONCLUSION

The findings of this study suggest that an AFO may be an acceptable therapeutic device to improve physical activity in participants with PAD, especially if appropriate improvements that promote adherence to its use are implemented. Future research should explore the long-term adherence to assistive devices in participants with PAD as well as evaluate different types of AFOs in a population with PAD.

## Figures and Tables

**TABLE 1:** Sample interview questions

Construct	Questions
Acceptability/Implementation	Tell me about your experience using the AFO. How often do you wear the AFO? What stops you from wearing it? What would encourage you to wear it more often? Based on your previous experience with the AFO, how often do you plan on wearing it in the future? Why? What factors impact your use of the AFO?
Practicality	4. What did you notice after you started wearing the AFO? What positive impacts did you feel? What negative impacts did you feel?

AFO, ankle foot orthosis.

**TABLE 2:** Results of paired-samples *t*-test to examine differences between means at the midpoint and endpoint and for weekdays and weekends

	Midpoint mean (SD)	Endpoint mean (SD)	Mean difference (SD)	SEM	95% CI of the difference		*t*	Significance (two-tailed)
					Lower	Upper		
Total days	5.36 (1.86)	5.00 (2.05)	0.36 (1.69)	0.51	−0.77	1.50	0.71	.49
Total hours per day	7.63 (4.24)	7.09 (5.09)	0.54 (2.81)	0.85	−1.35	2.43	0.64	.54
Only weekdays	3.82 (1.60)	3.82 (1.47)	0.00 (1.48)	0.45	−1.00	1.00	0.00	>.99
Only weekday hours per day	8.77 (3.93)	7.74 (4.66)	1.03 (2.40)	0.73	−0.58	2.65	1.42	.19
Only weekend days	1.55 (0.70)	1.18 (0.87)	0.36 (0.81)	0.24	−0.18	0.91	1.49	.17
Only weekend hours per day	6.49 (5.02)	6.44 (5.99)	0.05 (4.23)	1.28	−2.80	2.89	0.04	.97

Abbreviations: CI, confidence interval; SD, standard deviation; SEM, standard error mean.

* Significance deemed at *p* < .05.

n = 11.
